# Isolation and Characterization of Novel Bacteria Capable of Degrading 1,4-Dioxane in the Presence of Diverse Co-Occurring Compounds

**DOI:** 10.3390/microorganisms9050887

**Published:** 2021-04-21

**Authors:** Tanmoy Roy Tusher, Takuya Shimizu, Chihiro Inoue, Mei-Fang Chien

**Affiliations:** 1 Graduate School of Environmental Studies, Tohoku University, Sendai 980–8579, Japan; trtusher.esrm@gmail.com (T.R.T.); takuya.shimizu.p3@dc.tohoku.ac.jp (T.S.); chihiro.inoue.b1@tohoku.ac.jp (C.I.); 2 Department of Environmental Science and Resource Management, Mawlana Bhashani Science and Technology University, Santosh, Tangail-1902, Bangladesh

**Keywords:** 1,4-dioxane, biodegradation, metabolic degrader, SDIMOs, *Dokdonella*, carbon source, wastewater treatment

## Abstract

Biodegradation is found to be a promising, cost-effective and eco-friendly option for the treatment of industrial wastewater contaminated by 1,4-dioxane (1,4-D), a highly stable synthetic chemical and probable human carcinogen. This study aimed to isolate, identify, and characterize metabolic 1,4-D-degrading bacteria from a stable 1,4-D-degrading microbial consortium. Three bacterial strains (designated as strains TS28, TS32, and TS43) capable of degrading 1,4-D as a sole carbon and energy source were isolated and identified as Gram-positive *Pseudonocardia* sp. (TS28) and Gram-negative *Dokdonella* sp. (TS32) and *Afipia* sp. (TS43). This study, for the first time, confirmed that the genus *Dokdonella* is involved in the biodegradation of 1,4-D. The results reveal that all of the isolated strains possess inducible 1,4-D-degrading enzymes and also confirm the presence of a gene encoding tetrahydrofuran/dioxane monooxygenase (*thmA/dxmA*) belonging to group 5 soluble di-iron monooxygenases (SDIMOs) in both genomic and plasmid DNA of each of the strains, which is possibly responsible for the initial oxidation of 1,4-D. Moreover, the isolated strains showed a broad substrate range and are capable of degrading 1,4-D in the presence of additional substrates, including easy-to-degrade compounds, 1,4-D biodegradation intermediates, structural analogs, and co-contaminants of 1,4-D. This indicates the potential of the isolated strains, especially strain TS32, in removing 1,4-D from contaminated industrial wastewater containing additional organic load. Additionally, the results will help to improve our understanding of how multiple 1,4-D-degraders stably co-exist and interact in the consortium, relying on a single carbon source (1,4-D) in order to develop an efficient biological 1,4-D treatment system.

## 1. Introduction

1,4-dioxane (1,4-D), a synthetic chemical and probable human carcinogen, is widely used in various industrial units, e.g., as a solvent and chelating agent in chemical industries [1], as a purifying agent in pharmaceutical industries [2], as a wetting and dispersing agent in textile industries [3], and as a food additive in food industries [2]. It is also produced as an unintentional by-product during the manufacture of ethylene glycol (EG), ethylene oxide, detergents, and polyesters [1,2,3]. Owing to its extreme water solubility and low volatility from water, 1,4-D is commonly found in industrial wastewaters at high concentrations (up to several hundred mg/L) [4,5]. On the other hand, due to 1,4-D’s high physiochemical and biological stability, it is extremely difficult to remove it from industrial wastewater using most of the conventional physicochemical and biological treatment technologies [6]. Consequently, 1,4-D is frequently detected in the aquatic environments, where it remains persistent for a prolonged period of time [7,8,9]. Considering its potential carcinogenic effects [10], the World Health Organization (WHO) has listed 1,4-D as a ‘hazardous compound’ in WHO guidelines for drinking water [11], while the U.S. Environmental Protection Agency (USEPA) has listed it as a ‘high priority’ pollutant in the recent amendment to the Toxic Substances Control Act [12]. Therefore, 1,4-D has elicited great concern as an emerging water contaminant, and development of effective treatment technologies for 1,4-D-contaminated industrial wastewater is of paramount importance to mitigate subsequent ecological and human health risks.

Over the past decades, a plethora of phylogenetically diverse 1,4-D metabolizing and co-metabolizing bacteria have been isolated and identified [6,13,14,15,16]. As a result, biological treatment using 1,4-D-degrading bacteria has been receiving substantial attention as it is cost effective, energy efficient, and environment friendly as compared to advanced oxidation processes (AOPs), the only available chemical treatment technologies for 1,4-D [13,17]. Soluble di-iron monooxygenases (SDIMOs), a family of multicomponent bacterial enzymes, have been reported to play essential role in the metabolic and co-metabolic 1,4-D biodegradation [18,19]. SDIMOs can be categorized into six groups depending on their substrate preference, sequence similarity, and gene component arrangement [20,21], and group 5 and 6 SDIMOs (tetrahydrofuran/dioxane and propane monooxygenases, respectively) have been reported as the key bacterial enzymes initiating the biodegradation of 1,4-D by metabolic 1,4-D-degraders [22]. To date, a few Gram-negative 1,4-D-degrading bacterial strains have been isolated and characterized, while most of the reported 1,4-D-degraders are Gram-positive bacterial strains, mostly belonging to the genera *Pseudonocardia*, *Mycobacterium*, and *Rhodococcus*, which result in low cell yields and 1,4-D-degradation rates as compared to Gram-negative degraders [16]. Additional carbon sources are required to increase the cell yields of Gram-positive degraders, leading to an increase in additional costs and environmental burden [23,24]. On the other hand, among the Gram-negative degraders, many of the isolated strains cannot degrade 1,4-D as a sole carbon and energy source [6,14,18,23].

1,4-D-contaminated industrial wastewater is usually mixed with other organic compounds ranging from easily degradable organic materials to recalcitrant organic co-contaminants [6,13,25,26,27], which makes biological treatment of 1,4-D-contaminated wastewater challenging. However, the effects of various co-existing organic compounds on the biodegradation of 1,4-D by metabolic 1,4-D-degrading strains have been rarely studied [26,27,28,29,30]. Thus, identification and characterization of novel 1,4-D-degrading bacteria, especially the Gram-negative degraders, capable of utilizing 1,4-D as a sole carbon and energy source, are important to assess their potential in the application of treatment of 1,4-D-contaminated industrial wastewater containing additional organic load. In our previous study [15], we enriched a stable 1,4-D-degrading microbial consortium, named N112, from industrial wastewater and assumed that the consortium would consist of novel Gram-negative 1,4-D-degraders coexisting with well-known 1,4-D-degrading Gram-positive bacteria belonging to the genus *Pseudonocardia*. Thus, this study aimed to isolate, identify, and characterize metabolic 1,4-D-degraders co-existing in the consortium N112 in order to investigate their 1,4-D degradation behaviors; key enzymes initiating 1,4-D degradation; the substrate range; and 1,4-D degradation efficacy in the presence of additional carbon sources, including easy-to-degrade compounds, potential biodegradation intermediates, structural analogs, and other organic co-contaminants.

## 2. Materials and Methods

### 2.1. Chemicals

All chemicals, reagents and solvents used in this study were reagent-grade and were purchased from Wako Pure Chemical Industries, Ltd. (Osaka, Japan). 

### 2.2. Isolation and Identification of Metabolic 1,4-D-Degrading Bacteria

The metabolic 1,4-D-degrading bacteria was isolated by spreading the microbial inoculum of the consortium N112 on mineral salt medium (MSM, pH 7.0) agar plates supplemented with 100 mg/L of 1,4-D. The composition of MSM used in this study is described in Tusher et al. [15]. After incubating the plates at 30 °C, single colonies of distinct bacterial species were identified and inoculated into 20 mL vials containing 10 mL of sterile MSM supplemented with 100 mg/L of 1,4-D. Unless otherwise stated, all the cultivations in liquid MSM were performed aerobically at 30 °C with rotary shaking at 170 rpm. 1,4-D concentration of each inoculated vial was measured regularly to check the 1,4-D degradation ability of the colonies as a sole carbon and energy source.

The isolated 1,4-D-degrading bacterial strains were identified by 16S ribosomal RNA (rRNA) gene sequencing. Briefly, genomic DNA (gDNA) was extracted from the isolated strains using DNA Purification Kit from Wizard Genomics (Promega, Madison, WI, USA) following the manufacturer’s instruction. The concentration of the extracted gDNA was determined using a nanophotometer (Model: C40; IMPLEN, Munich, Germany), while the quality was checked by agarose gel (1.5%) electrophoresis. The 16S rRNA genes were amplified by polymerase chain reaction (PCR) (Model: PCR System 9700, Applied Biosystems, Waltham, MA, USA) using the extracted gDNA and the primer set: 27F (5′-AGAGTTTGATCCTGGCTCAG-3′) and 1492R (5′-GGTTACCTTGTTACGACTT-3´) obtained from Eurofins Genomics Co., Ltd. (Tokyo, Japan). The PCR was performed under the following thermocycling profile: initial denaturation at 94 °C for 5 min followed by 30 cycles of denaturation at 94 °C for 30 s, annealing at 55 °C for 30 s, elongation at 72 °C for 90 s, and finally extension at 72 °C for 7 min. The 16S rRNA amplicon sequencing was then performed by Bioengineering Lab. Co., Ltd. (https://www.gikenbio.com, accessed on: 20 April 2021). The obtained 16S rRNA gene sequences were checked using CodonCode Alinger and compared with the reference sequences in the NCBI database using BLAST similarity searches (https://blast.ncbi.nlm.nih.gov/, accessed on: 20 April 2021) to identify the isolated 1,4-D-degrading strains. The 16S rRNA gene sequences of the closely related strains were also obtained from Genbank database (https://www.ncbi.nlm.nih.gov/genbank/, accessed on: 20 April 2021), and, finally, the phylogenetic trees were constructed by aligning all the sequences using MEGA X [31]. Gram staining was conducted using Bacterial Gram Color Kit containing crystal violet, lugol, safranin, and decolorizing solution.

### 2.3. Analytical Procedures

The concentrations of 1,4-D were measured using a 7820A Gas Chromatograph (GC) system equipped with flame ionization detector (FID) (Agilent Technologies, Inc., Santa Clara, CA, USA). The GC-FID was equipped with an HP-5 column (30 m × 0.32 mm i.d., 0.25 µm film thickness). Helium gas was used as the carrier gas. The column oven temperature was set at 50 °C, while the injector and detector temperatures were fixed at 200 °C and 230 °C, respectively. The frozen micro-extraction method was followed to extract and analyze 1,4-D [32]. Briefly, 0.7 mL cultured sample was filtered through a membrane filter (Millex-GV, pore size: 0.22 µm, Merck Millipore, Burlington, MA, USA), and, then, 0.5 mL of the filtered sample was transferred to a 2 mL amber vial (Thermo Fisher Scientific Inc., Rockwood, TN, USA) containing 0.5 mL of dichloromethane (DCM) used as a solvent. The capped vial containing the reaction mixture was then vigorously shaken for 30 s to extract the 1,4-D with the DCM solvent phase. After freezing at −80 °C for 20 min, the liquid DCM was quickly transferred from the vial to a fresh vial. Finally, 2 µL of the extracted liquid was injected into GC-FID by a gas tight syringe (Hamilton Company Inc., Reno, NV, USA) to measure the 1,4-D concentration.

### 2.4. Preparation of Inocula of Isolated Strains for 1,4-D Degradation Experiments

Unless otherwise stated, to prepare the inoculum of the 1,4-D-degrading bacteria for 1,4-D degradation experiments, each of the isolated strains was cultured for 10 days in 120 mL vials containing 50 mL of sterile MSM supplemented with 100 mg/L of 1,4-D or glucose. The cells were harvested by centrifugation (10,000× *g*, 10 min, 4 °C) and washed twice with sterile MSM. The washed cells were then resuspended into fresh sterile MSM to prepare dense cell suspension, which was used as inoculum to perform all of the 1,4-D degradation experiments. Unless otherwise stated, abiotic control without inoculation was prepared for all the degradation experiments, while the experiments were conducted in duplicates.

### 2.5. Evaluation of 1,4-D-Degrading Enzyme Inducibility

To assess whether the 1,4-D-degrading enzymes associated with the isolated strains are inducible or constitutive, 1,4-D degradation experiments were performed with cells precultivated with 100 mg/L of 1,4-D or glucose. Afterwards, inducibility of the 1,4-D-degrading enzymes was evaluated by comparing the 1,4-D degradation profiles of the cells pre-grown on 1,4-D with those pre-grown on glucose.

### 2.6. Detection and Localization of Involed SDIMO Genes

The presence of SDIMO genes in both gDNA and plasmids of the isolated strains was checked by PCR using the degenerate primer set NVC57 (5′-CAGTCNGAYGARKCSCGNCAYAT-3′) and NVC66 (5′-CCANCCNGGRTAYTTRTTYTCRAACCA-3′) targeting the conserved sequence of SDIMO α-subunit [20]. Each 25 µL of PCR mixture contained 2.5 μL of 10× Ex Taq Buffer, 2.0 μL of dNTPs (2.5 mM), 2.5 µL of each primer (10 µM), 0.2 µL of Ex Taq HS polymerase (5 U/µL), 0.5 µL of Mg^2+^ (25 mM), and 3.0 µL of gDNA (10–20 ng) as template. The Ex Taq Buffer, dNTPs, and Ex Taq polymerase were purchased from TaKaRa Bio Inc. (Shiga, Japan). The PCR was performed under the following thermocycling conditions: initial denaturation at 94 °C for 5 min followed by 30 cycles of denaturation at 94 °C for 30 s, annealing at 60 °C for 30 s, elongation at 72 °C for 1 min, and finally extension at 72 °C for 5 min. The PCR amplicons were checked for the correct product size by agarose gel (1.5%) electrophoresis and then gel-purified using NucleoSpin® Gel and PCR Clean-up Kit (Macherey-Nagel GmbH & Co. KG, Düren, Germany). The PCR amplified products were then ligated into the pMD19-T vector and transformed into *E. coli* JM109 competent cells using standard protocol [33]. Positive recombinant plasmids were selected by the white/blue screening method and identified by colony PCR. The plasmid-SDIMO DNA was extracted and purified from *E. coli* JM109 cells, and, finally, the sequencing was performed by Bioengineering Lab. Co., Ltd. The obtained SDIMO gene sequences were checked using CodonCode Alinger, and a phylogenetic tree was constructed by aligning with the amino acid sequences of other SDIMO α-subunit genes obtained from the Genbank database using MEGA X [31].

### 2.7. Evaluation of Substrate Range 

To evaluate the ability of the isolated 1,4-D-degrading bacteria to utilize various organic compounds, the growth of each of the bacterial strains on eight different organic compounds, in addition to 1,4-D, including glucose, lactic acid (LA), EG, tetrahydrofuran (THF), phenol, tetradecane (TD), toluene, and 1,1,1-trichloroethane (1,1,1-TCA), was investigated. Briefly, the isolated 1,4-D-degrading strains were cultured in 50 mL vials containing 20 mL sterile MSM supplemented with no substrate or one of the tested organic substrates (100 mg-C/L). After culturing for 7 days, the biomass was collected by centrifugation (13,000× *g*, 10 min, 4 °C), and gDNA was extracted using DNA Purification Kit from Wizard Genomics (Promega, Madison, WI, USA), following the manufacturer’s protocol. The concentration and quality of the extracted gDNA were determined using a nanophotometer (Model: C40; IMPLEN, Munich, Germany) and agarose gel (1.5%) electrophoresis, respectively. The cell growth was then quantified as a measure of 16S rRNA gene copy numbers/mL by quantitative real time PCR (qRT-PCR) on a CFX Connect^TM^ Real-time System (Bio-Rad, Hercules, CA, USA) using the 341F (5′-CCTACGGGAGGCAGCAG-3′) and 518R (5′-ATTACCGCGGCTGCTGG-3′) primer set under the following thermocycling profile: 95 °C for 30 s followed by 40 cycles of at 95 °C for 5 s, 57 °C for 30 s, and 72 °C for 30 s. Each 15 μL of qRT-PCR solution contained 7.5 μL of TB Green® premix Ex Tag^TM^ II (TaKaRa Bio Inc., Shiga, Japan), 0.3 µL of each primer (10 µM), and 2.0 µL of gDNA (<100 ng) as template. Melting curve analysis was performed by heating the PCR products from 55 °C to 95 °C at a transition rate of 0.5 °C/s. The Milli-Q water was used as the negative control, while all the qRT-PCR amplifications were conducted in duplicates. The standard for quantifying the 16S rRNA gene copy numbers was prepared with purified plasmid DNA containing the target region of 16S rRNA, and the standard curve (R^2^ > 0.99) was generated from the amplifications of serially diluted plasmid DNA (10^8^–10^4^/µL).

### 2.8. 1,4-D Degradation Experiments in the Presence of Additional Carbon Sources

To investigate the 1,4-D biodegradability of the isolated strains in the presence of additional carbon sources, all the organic compounds used for substrate utilization experiments were investigated. In this regard, glucose and LA were considered easy-to-degrade carbon sources, while other compounds (EG, THF, phenol, TD, toluene, and 1,1,1-TCA) were considered organic co-contaminants of 1,4-D. Moreover, EG is known as a potential 1,4-D biodegradation intermediate [34], whereas THF is a structural analog of 1,4-D [1]. Briefly, the isolated 1,4-D-degrading strains were cultured in 50 mL vials containing 20 mL sterile MSM supplemented with 1,4-D (100 mg/L) and one of the additional organic compounds (100 mg-substrate/L). The degradation experiments were conducted for 19 days, while samples were regularly collected for monitoring the biodegradation of 1,4-D. The biodegradation efficiency was calculated using the following equation:

(1) $$\;\mathrm{Biodegradation}\;\mathrm{efficiency}\;\;(\%)\;=\;\frac{C_{0}-C_{t}}{C_{0}}\;\times \;100$$

where C_0_ and C_t_ represent the initial and final concentrations of 1,4-D (mg/L), respectively.

## 3. Results

### 3.1. Isolation and Identification of Metabolic 1,4-D-Degrading Bacteria

**Figure 1:**
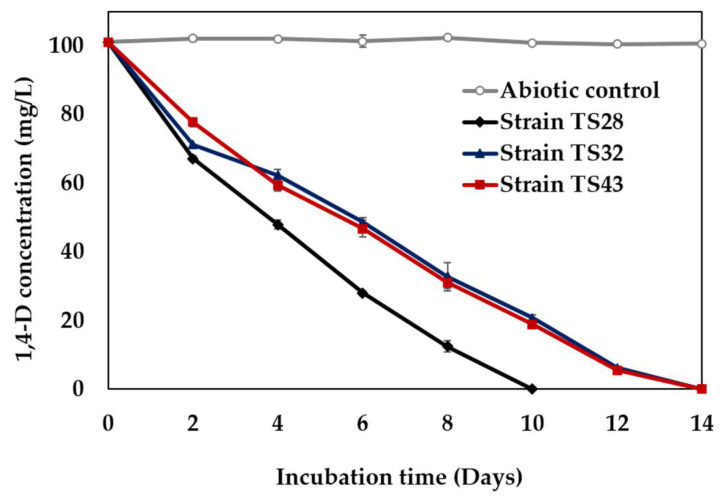
Typical 1,4-D (100 mg/L) degradation profiles of three isolated strains under aerobic conditions. Error bars represent the absolute mean deviations (MD) from the duplicate experiments and may be smaller than the markers.

Three bacterial strains (designated as strains TS28, TS32, and TS43) capable of degrading 1,4-D as a sole carbon and energy source were isolated from the stable consortium. Figure 1 shows the typical 1,4-D degradation profiles of the isolated metabolic 1,4-D-degrading bacterial strains, while no degradation occurred in abiotic controls. All of the isolated strains formed aggregation in liquid medium supplemented with 1,4-D (Figure S1a), while white colonies were observed when cultured onto MSM agar plates supplemented with 1,4-D (Figure S1b). Gram staining analysis revealed that the strain TS28 is a Gram-positive bacterium, while the strains TS32 and TS43 are Gram-negative (Figure S1c).

**Figure 2:**
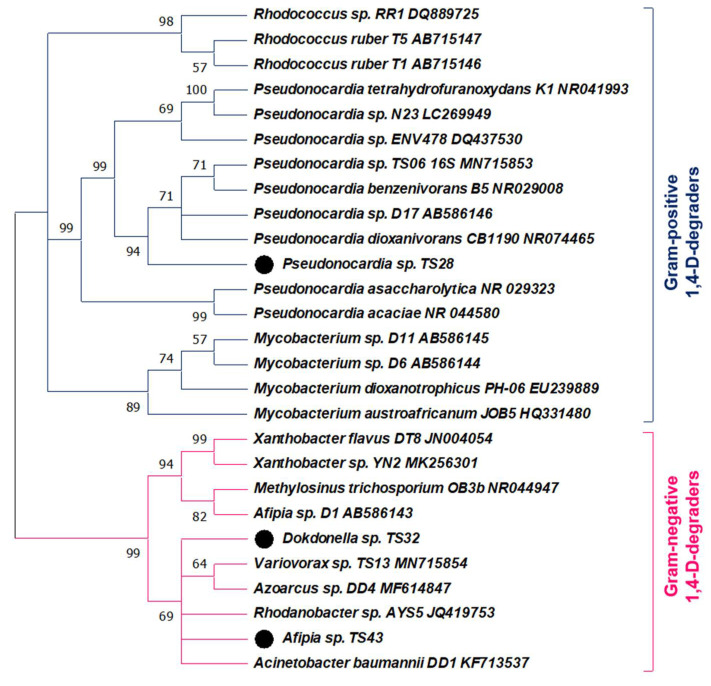
Neighbor-joining tree depicting phylogenetic relationships among 1,4-D-degrading strains isolated in this study (indicated by the black circles) and previously reported Gram-positive and Gram-negative 1,4-D-degraders. The nucleotide sequences of 16S rRNA genes were used to construct the phylogenetic tree, whereas the numbers at the branching points are the bootstrap values of 1000 replications.

The results of BLAST similarity searches based on the partial 16S rRNA gene sequences showed that the strains TS28, TS32 and TS43 are closely related to *Pseudonocardia* spp., *Dokdonella* spp., and *Afipia* spp., respectively. The 16S rRNA gene sequence of the strain TS28 was 100% (of 1334 bp) identical to the previously reported Gram-positive metabolic 1,4-D-degrader *Pseudonocardia dioxanivorans* CB1190 (NR074465). On the other hand, the 16S rRNA gene sequence of the strain TS32 was 99.37% (of 1273 bp) identical to that of an aromatic hydrocarbon-degrading bacterium *Dokdonella* sp. TSY06 (AB663505) belonging to the class Gammaproteobacteria. The 16S rRNA gene sequence of the strain TS43 was 100% (of 1391) identical to *Afipia* sp. SP3302452 (AY599913) belonging to the class Alphaproteobacteria. Therefore, the isolated strains were identified as *Pseudonocardia* sp. TS28, *Dokdonella* sp. TS32, and *Afipia* sp. TS43. The partial sequences of 16S rRNA genes of the isolated strains are available in the GenBank database under the accession numbers MW805251 (strain TS28), MW805252 (strain TS32), and MW805253 (strain TS43). Further phylogenetic analysis revealed that the strains TS32 and TS43 are evolutionarily distinct from the Gram-negative 1,4-D-degrading bacteria identified previously (Figure 2).

### 3.2. Inducibility of 1,4-D-Degrading Enzymes

**Figure 3:**
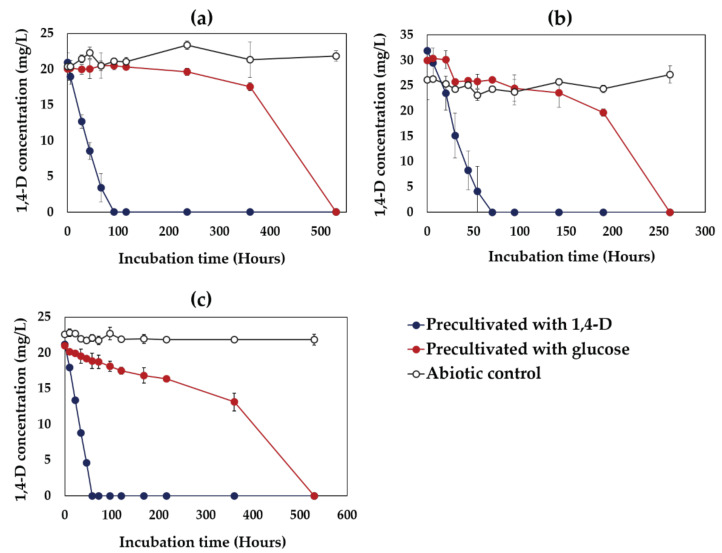
Inducibility of 1,4-D-degrading enzymes in the strains TS28 (**a**), TS32 (**b**), and TS43 (**c**). The initial 1,4-D and cell concentration was 20 mg/L and 394 mg-fresh cell/L, respectively. Error bars represent the MD from the duplicate experiments and may be smaller than the markers.

When the cells precultivated with 1,4-D were used to degrade 20 mg/L of 1,4-D, complete degradation of 1,4-D by all the strains was observed without a lag phage, while no 1,4-D degradation occurred in abiotic controls (Figure 3). On the other hand, when the cells precultivated with glucose were used in the same 1,4-D degradation experiments, 1,4-D degradation profiles differed greatly with a lag phase as compared to that observed with the cells precultivated with 1,4-D (Figure 3). These results confirm that all of the 1,4-D-degrading bacteria isolated in this study possess inducible 1,4-D-degrading enzymes.

### 3.3. Detection and Localization of SDIMO Genes in the Isolated Strains

**Figure 4:**
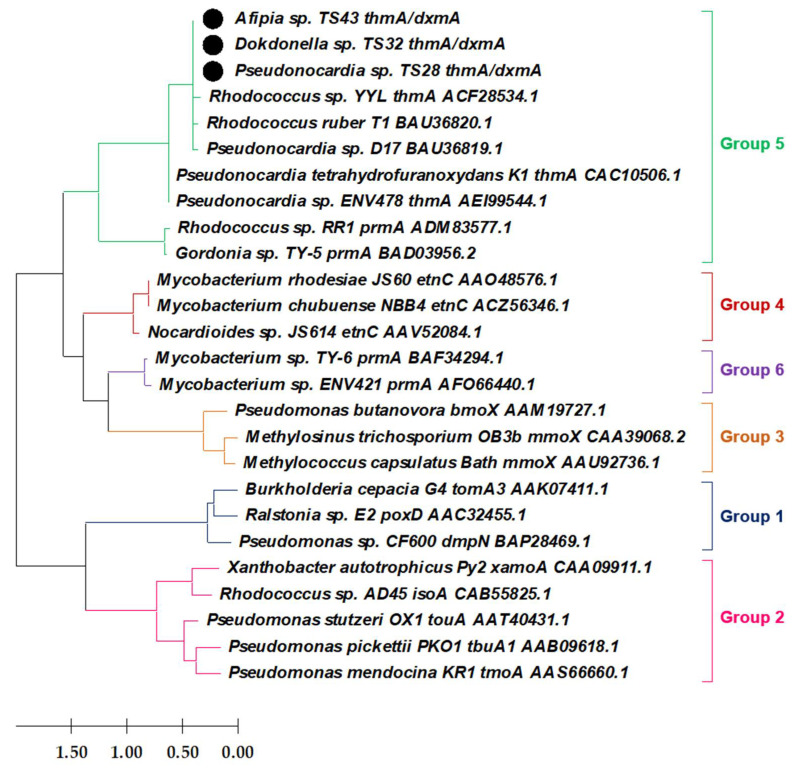
Phylogenetic relationships of SDIMO α-subunit genes (i.e., *thmA/dxmA*) present on the chromosomes of the isolated strains (indicated by the black circles) and other SDIMO α-subunit genes as generated by the maximum likelihood method based on the alignment of amino acid sequences.

The PCR and gel electrophoresis results confirm that 1,4-D-degrading SDIMO genes are located on both chromosomes and plasmids of all the isolated strains (data not shown). The sub-cloning followed by sequencing of the chromosomally encoded SDIMO genes deciphered that these three isolated 1,4-degrading bacteria possess the same SDIMO gene on the chromosome. Finally, the phylogenetic analysis revealed that the detected gene encodes the α-subunit of a THF/dioxane monooxygenase (*thmA/dxmA*) belonging to the group 5 SDIMOs (Figure 4), which is possibly the key enzyme initiating the degradation of 1,4-D by the metabolic 1,4-D-degraders isolated in this study. Further investigation also demonstrated the presence of *thmA/dxmA* gene in the plasmid DNA extracted from each of the isolated strains, which were 100% identical to the *thmA/dxmA* genes detected on the chromosomes of the isolated strains. The partial nucleotide sequences of the chromosomally encoded SDIMO α-subunit genes of the isolated strains were 100% identical to those of the THF monooxygenase α-subunit gene (*thmA*) of *Rhodococcus* sp. YYL and SDIMO α-subunit genes of *Pseudonocardia* sp. D17 and *Rhodococcus ruber* T1 (Figure 4). The partial sequences of *thmA/dxmA* genes detected in the genomic DNA of the isolated strains were deposited in the GenBank database under the accession numbers MW882968-MW882970.

### 3.4. Substrate Range of the Isolated Strains

**Figure 5:**
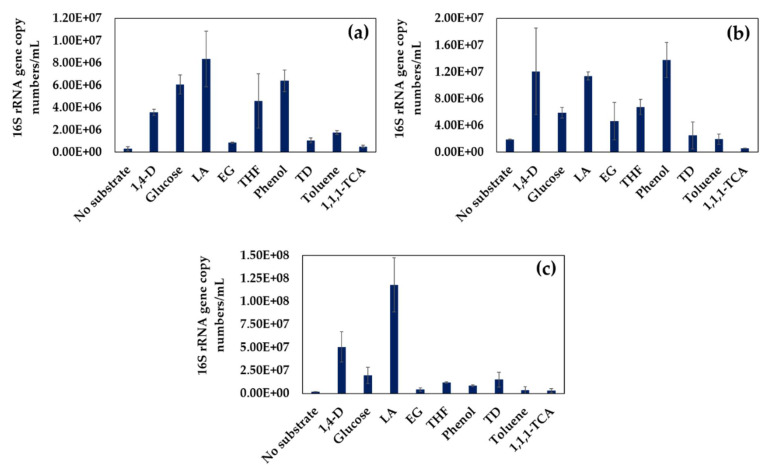
Ability of the strains TS28 (**a**), TS32 (**b**), and TS43 (**c**) to utilize various organic compounds (100 mg-C/L) as a growth substrate. Data shown are the cell growth measured as 16S rRNA gene copy numbers/mL after 7-day incubation experiments. The initial cell concentration was 100, 134, and 238 mg-fresh cell/L for strain TS28, TS32, and TS43, respectively. Error bars represent the MD from the duplicate experiments.

In addition to 1,4-D, all of the isolated strains were found to be capable of utilizing various substrates including easy-to-degrade compounds and co-contaminants of 1,4-D as sole carbon and energy sources (Figure 5). Significant growth of all the strains was observed when cultivated with glucose and LA, which are readily degradable by most of the bacteria. All of the isolated strains, especially TS28 and TS32, utilized EG (potential 1,4-D biodegradation intermediate), THF (structural analog of 1,4-D), phenol, and TD for their growth. However, the strains TS32 and TS43 were unable to grow on toluene or 1,1,1-TCA (Figure 5b,c), while strain TS28 was found to be able to grow on toluene (Figure 5a). The results indicate that the isolated 1,4-D-degrading strains have a broad substrate range.

### 3.5. 1,4-D Degradation Efficiency of the Isolated Strains in the Presence of Additional Carbon Source

The 1,4-D degradation efficiency (%) of each of the isolated metabolic 1,4-D-degraders in the presence of various easy-to-degrade compounds (glucose and LA) and organic co-contaminants (EG, THF, phenol, TD, toluene, and 1,1,1-TCA) is presented in Table 1. Moreover, the detailed 1,4-D degradation profiles of the isolated strains in the presence of tested substrates are depicted in Figure S2. After 12 days of incubation, the strain TS32 degraded 100% of the initial 1,4-D concentration (100 mg/L) in the absence of additional substrate, while about 85% and 95% 1,4-D degradation was caused by the strains TS28 and TS43, respectively (Table 1). No noticeable 1,4-D degradation occurred in abiotic controls after 19 days of incubation experiments (Figure S2).

The presence of glucose or LA slightly slowed down the 1,4-D degradation by the isolated strains and subsequently reduced the biodegradation efficiency (Figure S2). However, the novel strain TS32 was found to be more effective in degrading 1,4-D in the presence of easy-to-degrade compounds (Table 1). On the other hand, no significant effect of EG on the 1,4-D degradation was observed during the 19 days of incubation experiments (Figure S2). On the contrary, the co-occurrence of THF significantly inhibited the 1,4-D degradation (Figure S2), resulting in reduced 1,4-D biodegradation efficiency of the isolated strains: 4%, 14%, and 19% for TS28, TS43 and TS32, respectively (Table 1). As compared to THF, the co-occurrence of phenol, TD, or 1,1,1-TCA resulted in relatively higher 1,4-D degradation, although inhibition in 1,4-D degradation was observed when compared with the 1,4-D degradation profiles in the absence of additional compounds (Figure S2). As compared to others, the 1,4-D degradation efficacy of the strain TS32 was found to be superior in the presence of these organic co-contaminants (phenol, TD, and 1,1,1-TCA) (Table 1). However, the co-existence of toluene significantly affected the 1,4-D degradation by the strain TS32 and resulted in only 9% 1,4-D degradation after 12 days, whereas about 24% and 16% 1,4-D degradation was caused by the strains TS28 and TS43, respectively (Table 1). The results show that each of the isolated strains have the ability to degrade 1,4-D in the presence of diverse co-occurring organic compounds, while the novel strain TS32 seems to be highly efficient and robust for the treatment of 1,4-D-contaminated industrial wastewater.

## 4. Discussion

Isolation and characterization of metabolic 1,4-D-degrader is of utmost importance not only to develop an efficient biological treatment system using pure culture for 1,4-D-contaminated industrial wastewater [13,16] but also to understand their interactions while co-existing in the 1,4-D-degrading microbial consortia known to be more effective and robust as compared to pure culture [15]. The present study isolated and identified three metabolic 1,4-D-degrading bacteria (*Pseudonocardia* sp. TS28, *Dokdonella* sp. TS32, and *Afipia* sp. TS43) from a stable 1,4-D-degrading microbial consortium (Figure 1 and Figure 2). The Gram-positive bacteria belonging to the genus *Pseudonocardia* are well-known for their ability to degrade 1,4-D metabolically or co-metabolically and are commonly found in 1,4-D-degrading microbial consortia or mixed cultures enriched from different contaminated environments [1,35,36,37]. Until now, seven *Pseudonocardia* spp. capable of degrading 1,4-D metabolically, including strain TS28, have been identified [6,13,15,38].

In contrast, to date, six metabolic 1,4-D-degrading Gram-negative bacteria belonging to five genera have been identified: *Acinetobacter baumannii* DD1 [39], *Afipia* sp. D1 [38], *Rhodanobacter* sp. AYS5 [28], *Variovorax* sp. TS13 [15], *Xanthobacter* sp. YN2 [16], and *Xanthobacter flavus* DT8 [40]. In this study, two novel metabolic 1,4-D-degrading Gram-negative bacteria (strains TS32 and TS43) were identified. Although several studies observed the dominance of the genus *Dokdonella* in 1,4-D-degrading microbial consortia or enriched cultures [15,41,42], no 1,4-D-degrading bacterial strain belonging to the genus *Dokdonella* was reported previously. Therefore, this is the first study that successfully isolated and identified one *Dokdonella* sp. capable of degrading 1,4-D and confirmed the involvement of the genus *Dokdonella* in the biodegradation of 1,4-D. On the other hand, another Gram-negative strain TS43 belonging to the genus *Afipia* was found to be phylogenetically distinct from the previously identified 1,4-D-degrader *Afipia* sp. D1 [38].

The 1,4-D-degrading bacteria can be categorized into two groups, inducible and constitutive, depending on their ability to express 1,4-D-degrading enzymes [38]. In this study, all the isolated 1,4-D-degrading bacteria were found to have inducible 1,4-D-degrading enzymes (Figure 3). Several studies also reported that 1,4-D-degraders belonging to the genera *Pseudonocardia* and *Afipia* possess inducible 1,4-D-degrading enzymes [13,38]. However, it was also observed that the characteristics and mechanisms of 1,4-D degradation can vary from one 1,4-D-degrading strain to another, even though they belong to the same genus. For instance, *Pseudonocardia dioxanivorans* CB1190 possesses an inducible 1,4-D-degrading enzyme, while *Pseudonocardia* sp. D17 and *Pseudonocardia* sp. N23 possess constitutive 1,4-D-degrading enzymes [13,38].

Moreover, all of the 1,4-D-degrading bacteria isolated in this study possess identical *thmA/dxmA* genes belonging to group 5 SDIMOs in both genomic and plasmid DNA (Figure 4). SDIMOs are known for their ability to catalyze the initial oxidation of a wide range of pollutants, including aromatic hydrocarbons, alkanes, and alkenes [20]. The THF monooxygenases are the most common 1,4-D-degrading bacterial enzymes identified in metabolic 1,4-D-degraders, which catalyze the α-hydroxylation by inserting a hydroxyl group at the α-carbon position of 1,4-D, leading to the cleavage of a high-energy C-O bond [34,43,44,45]. Previous studies reported that the abundance of genes encoding *thmA/dxmA* was positively correlated with the degradation of 1,4-D [46], implying that *thmA* is possibly the key enzymes initiating 1,4-D degradation by the isolated metabolic 1,4-D-degraders. This is the first study that identified the responsible SDIMO group (group 5 *thmA/dxmA*) in the metabolic 1,4-D-degraders belonging to the genera *Dokdonella* and *Afipia*. In addition, the presence of genes encoding *thmA/dxmA* in the isolated strains is found to be located on the chromosomes, indicating that there is no risk of losing the 1,4-D degradation ability of the isolated strains as a result of plasmid curing that might have happened for the 1,4-D-degraders with SDIMO genes on plasmids [47,48]. Since the strains TS28, TS32, and TS43 are phylogenetically distinct, the same SDIMO gene owned by these isolates indicated that a possible horizontal gene transfer occurred in the consortium. Therefore, further investigation on the localization of these SDIMO genes would help to shed light on this point.

Furthermore, the 1,4-D-degrading strains showed broad substrate range, as they were found to be capable of utilizing a wide variety of substrates, including easy-to-degrade compounds, structural analogs, and organic co-contaminants of 1,4-D (Figure 5). The isolated strains were found to grow on EG, a potential 1,4-D biodegradation intermediate, suggesting that the isolated strains may degrade 1,4-D following the biodegradation pathway through EG, as proposed previously for *P. dioxanivorans* CB1190 possessing THF monooxygenase [34,45]. However, further study is required to confirm the 1,4-D biodegradation pathway of the isolated strains. On the other hand, it was found that 1,4-D-degrading bacteria can utilize THF for their growth [13,16,38,39,40,43], which also supports the results of the present study. The isolated strains can also grow on recalcitrant organic compounds, such as phenol and TD [49], while strains TS32 and TS43 were unable to utilize toluene or 1,1,1-TCA (Figure 5).

Biodegradation of 1,4-D can be retarded in the co-occurrence of other organic compounds, including easily degradable and recalcitrant compounds [25,26,28,50]. Interestingly, the 1,4-D-degrading bacteria isolated in this study showed a great potential to degrade 1,4-D in the presence of diverse co-occurring organic compounds, while *Dokdonella* sp. TS32 was found to be highly efficient among others (Table 1 and Figure S2). However, the presence of glucose and LA slightly inhibited 1,4-D degradation, which might be due to the consumption of these easy-to-degrade compounds by bacteria rather than 1,4-D. Similar results were also reported by Pugazhendi et al. [28]. On the other hand, the co-occurrence of EG did not show any significant effects on the 1,4-D degradation profiles, which might be attributed to the low initial EG concentration (100 mg/L), and the result was found to be similar to that reported by Sei et al. [38]. In contrast, the co-occurrence of THF significantly inhibited the 1,4-D degradation, which was possible due to the competitive inhibition as also reported by other studies [25,50]. 1,4-D degrading enzymes probably have higher affinity to THF than 1,4-D, which might be the reason behind such competitive inhibition [25,26]. Substantial inhibition also occurred in the presence of toluene. However, such inhibition with toluene was not competitive but possibly resulted from the toxic effects of toluene on bacteria [51]. The presence of phenol, a preferred growth substrate for the isolated strains (Figure 5), also inhibited the degradation of 1,4-D, possibly due to the preferential degradation of phenol rather than 1,4-D by the bacteria, while the co-existence of TD and 1,1,1-TCA resulted in relatively less inhibition, which might be due to the low substrate concentrations and chemical interactions with the 1,4-D-degrading monooxygenase enzymes [29].

## 5. Conclusions

The study isolated three metabolic 1,4-D-degrading bacteria possessing inducible 1,4-D-degrading enzymes. Among them, *Dokdonella* sp. TS32 and *Afipia* sp. TS43 are newly identified 1,4-D-degraders, which are phylogenetically distinct from the previously identified Gram-negative 1,4-D-degrading bacteria. This study, for the first time, confirmed the involvement of the genus *Dokdonella* in the biodegradation of 1,4-D. All the isolated strains possess identical genes encoding *thmA/dxmA* belonging to group 5 SDIMOs, which is possibly the responsible enzyme initiating the degradation of 1,4-D. The isolated strains are capable of utilizing various substrates and also showed the ability to degrade 1,4-D in the presence of diverse co-occurring compounds ranging from easy-to-degrade compounds to recalcitrant organic co-contaminants. The findings clearly indicate that the isolated strains, especially *Dokdonella* sp. TS32, have a great potential to be applied for the treatment of 1,4-D-contaminated industrial wastewater containing diverse co-occurring compounds. Additionally, the results of this study will help to improve our understanding of how these metabolic 1,4-D-degraders stably co-exist and interact in the 1,4-D-degrading consortium N112, relying on 1,4-D as the only carbon and energy source in order to develop an efficient biological 1,4-D treatment system.

## Figures and Tables

**Table 1:** 1,4-D degradation efficiency of the isolated strains in the presence of additional carbon sources (100 mg-substrate/L) after 12 days of incubation.

Substrates	1,4-D Degradation Efficiency (%)		
	Strain TS28	Strain TS32	Strain TS43
1,4-D only	85.3 ± 5.66	100.0 ± 0.00	94.6 ± 7.60
1,4-D + glucose	85.7 ± 0.02	72.6 ± 0.13	67.6 ± 2.34
1,4-D + LA	77.6 ± 12.69	90.0 ± 14.16	59.0 ± 0.45
1,4-D + EG	90.8 ± 6.41	99.0 ± 0.25	88.0 ± 1.75
1,4-D + THF	4.4 ± 0.94	18.5 ± 12.11	14.0 ± 5.33
1,4-D + phenol	55.4 ± 1.70	53.0 ± 1.60	43.7 ± 3.31
1,4-D + TD	62.5 ± 16.91	92.3 ± 4.43	65.1 ± 3.04
1,4-D + toluene	24.0 ± 0.54	8.7 ± 5.58	15.7 ± 4.76
1,4-D + 1,1,1-TCA	63.9 ± 0.36	66.5 ± 2.50	63.8 ± 6.38

The initial 1,4-D and cell concentration was 100 mg/L and 162 mg-fresh cell/L, respectively, for all the experiments. Data shown are the mean ± MD from the duplicate experiments.

## Data Availability

Not applicable.

## Supplementary Materials

The following are available online at https://www.mdpi.com/article/10.3390/microorganisms9050887/s1, Figure S1: (a) Photographs of the isolated 1,4-D-degrading strains forming aggregation in the liquid medium supplemented with 1,4-D, (b) colonies of isolated strains on MSM agar plate supplemented with 1,4-D, and (c) Gram staining of the isolated strains; Figure S2: Degradation of 1,4-D in the presence of additional carbon sources (100 mg-substrate/L) by isolated strains TS28, TS32, and TS43 under aerobic conditions at 30 °C and 170 rpm.

## References

[1] Inoue D., Hisada K., Okumura T., Yabuki Y., Yoshida G., Kuroda M., Ike M. (2019). Carbon sources that enable enrichment of 1,4-dioxane-degrading bacteria in landfill leachate. Biodegradation.

[2] Duncan B., Vavricka E., Morrison R. (2004). A forensic overview of 1,4-dioxane. Environ. Claims J..

[3] Zenker M.J., Borden R.C., Barlaz M.A. (2003). Occurrence and treatment of 1,4-dioxane in aqueous environments. Environ. Eng. Sci..

[4] Han J.S., So M.H., Kim C.G. (2009). Optimization of biological wastewater treatment conditions for 1,4-dioxane decomposition in polyester manufacturing processes. Water Sci. Technol..

[5] Barndōk H., Cortijo L., Hermosilla D., Negro C., Blanco Á. (2014). Removal of 1,4-dioxane from industrial wastewaters: Routes of decomposition under different operational conditions to determine the ozone oxidation capacity. J. Hazard. Mater..

[6] Zhang S., Gedalanga P.B., Mahendra S. (2017). Advances in bioremediation of 1,4-dioxane-contaminated waters. J. Environ. Manag..

[7] Stepien D.K., Diehl P., Helm J., Thomas A., Püttmann W. (2014). Fate of 1,4-dioxane in the aquatic environment: From sewage to drinking water. Water Res..

[8] Adamson D.T., Mahendra S., Walker K.L., Rauch S.R., Sengupta S., Newell C.J. (2014). A multisite survey to identify the scale of the 1,4-dioxane problem at contaminated groundwater sites. Environ. Sci. Technol. Lett..

[9] Adamson D.T., de Blanc P.C., Farhat S.K., Newell C.J. (2016). Implications of matrix diffusion on 1,4-dioxane persistence at contaminated groundwater sites. Sci. Total Environ..

[10] IARC (International Agency for Research on Cancer) (1999). Re-evaluation of some organic chemicals, hydrazine and hydrogen peroxide. IARC Monographs on the Evaluation of Carcinogenic Risks to Humans.

[11] WHO (World Health Organization) (1993). Guidelines for Drinking-Water Quality.

[12] USEPA (U.S. Environmental Protection Agency) (2016). Statistics for the New Chemicals Review Program under TSCA.

[13] Yamamoto N., Saito Y., Inoue D., Sei K., Ike M. (2018). Characterization of newly isolated *Pseudonocardia* sp. N23 with high 1,4-dioxane-degrading ability. J. Biosci. Bioeng..

[14] Deng D., Li F., Wu C., Li M. (2018). Synchronic biotransformation of 1,4-dioxane and 1,1-dichloroethylene by a gram-negative propanotroph *Azoarcus* sp. DD4. Environ. Sci. Technol. Lett..

[15] Tusher T.R., Shimizu T., Inoue C., Chien M.-F. (2020). Enrichment and analysis of stable 1,4-dioxane-degrading microbial consortia consisting of novel dioxane-degraders. Microorganisms.

[16] Ma F., Wang Y., Yang J., Guo H., Su D., Yu L. (2021). Degradation of 1,4-dioxane by *Xanthobacter* sp. YN2. Curr. Microbiol..

[17] Kim C.G., Seo H.J., Lee B.R. (2006). Decomposition of 1,4-dioxane by advanced oxidation and biochemical process. J. Environ. Sci. Health A Toxic Hazard. Subst. Environ. Eng..

[18] Mahendra S., Alvarez-Cohen L. (2006). Kinetics of 1,4-dioxane biodegradation by monooxygenase-expressing bacteria. Environ. Sci. Technol..

[19] Gedalanga P.B., Pornwongthong P., Mora R., Chiang S.-Y.D., Baldwin B., Ogles D., Mahendra S. (2014). Identification of biomarker genes to predict biodegradation of 1,4-dioxane. Appl. Environ. Microbiol..

[20] Coleman N.V., Bui N.B., Holmes A.J. (2006). Soluble di-iron monooxygenase gene diversity in soils, sediments and ethene enrichments. Environ. Microbiol..

[21] Leahy J.G., Batchelor P.J., Morcomb S.M. (2006). Evolution of the soluble diiron monooxygenases. FEMS Microbiol. Rev..

[22] Li F., Deng D., Li M. (2020). Distinct catalytic behaviors between two 1,4-dioxane-degrading monooxygenases: Kinetics, inhibition, and substrate range. Environ. Sci. Technol..

[23] Sun B., Ko K., Ramsay J.A. (2011). Biodegradation of 1,4-dioxane by a Flavobacterium. Biodegradation.

[24] Rolston H.M., Hyman M.R., Semprini L. (2019). Aerobic cometabolism of 1,4-dioxane by isobutene-utilizing microorganisms including *Rhodococcus rhodochrous* strain 21198 in aquifer microcosms: Experimental and modeling study. Sci. Total Environ..

[25] Lee K.H., Wie Y.M., Jahng D., Yeom I.T. (2020). Effects of additional carbon sources in the biodegradation of 1,4-dioxane by a mixed culture. Water.

[26] Inoue D., Tsunoda T., Yamamoto N., Ike M., Sei K. (2018). 1,4-dioxane degradation characteristics of *Rhodococcus aetherivorans* JCM 14343. Biodegradation.

[27] Zhou Y., Huang H., Shen D. (2016). Multi-substrate biodegradation interaction of 1,4-dioxane and BTEX mixtures by *Acinetobacter baumannii* DD1. Biodegradation.

[28] Pugazhendi A., Banu J.R., Dhavamani J., Yeom I.T. (2015). Biodegradation of 1,4-dioxane by *Rhodanobacter* AYS5 and the role of additional substrates. Ann. Microbiol..

[29] Zhang S., Gedalanga P.B., Mahendra S. (2016). Biodegradation kinetics of 1,4-dioxane in chlorinated solvent mixtures. Environ. Sci. Technol..

[30] Chen D.-Z., Jin X.-J., Chen J., Ye J.-X., Jiang N.-X., Chen J.-M. (2016). Intermediates and substrate interaction of 1,4-dioxane degradation by the effective metabolizer *Xanthobacter flavus* DT8. Int. Biodeter. Biodegr..

[31] Kumar S., Stecher G., Li M., Knyaz C., Tamura K. (2018). MEGA X: Molecular evolutionary genetics analysis across computing platforms. Mol. Biol. Evol..

[32] Li M., Conlon P., Fiorenza S., Vitale R.J., Alvarez P.J.J. (2011). Rapid analysis of 1,4-dioxane in groundwater by frozen micro-extraction with gas chromatography/mass spectrometry. Groundwater Monit. Rem..

[33] Sambrook J., Russell D.W. (2001). Molecular Cloning: A Laboratory Manual.

[34] Grostern A., Sales C.M., Zhuang W.-Q., Erbilgin O., Alvarez-Cohen L. (2012). Glyoxylate metabolism is a key feature of the metabolic degradation of 1,4-dioxane by *Pseudonocardia dioxanivorans* strain CB1190. Appl. Environ. Microbiol..

[35] Xiong Y., Mason O.U., Lowe A., Zhou C., Chen G., Tang Y. (2019). Microbial community analysis provides insights into the effects of tetrahydrofuran on 1,4-dioxane biodegradation. Appl. Environ. Microbiol..

[36] Xiong Y., Mason O.U., Lowe A., Zhang Z., Zhou C., Chen G., Villalonga M.J., Tang Y. (2020). Investigating promising substrates for promoting 1,4-dioxane biodegradation: Effects of ethane and tetrahydrofuran on microbial consortia. Biodegradation.

[37] Inoue D., Yoshikawa T., Okumura T., Yabuki Y., Ike M. (2021). Treatment of 1,4-dioxane-containing water using carriers immobilized with indigenous microorganisms in landfill leachate treatment sludge: A laboratory-scale reactor study. J. Hazard. Mater..

[38] Sei K., Miyagaki K., Kakinoki T., Fukugasako K., Inoue D., Ike M. (2013). Isolation and characterization of bacterial strains that have high ability to degrade 1,4-dioxane as a sole carbon and energy source. Biodegradation.

[39] Huang H., Shen D., Li N., Shan D., Shentu J., Zhou Y. (2014). Biodegradation of 1,4-dioxane by a novel strain and its biodegradation pathway. Water Air Soil Pollut..

[40] Jin X.J., Chen D.Z., Zhu R.Y., Chen J., Chen J.M. (2012). Characteristics of 1,4-dioxane degradation by *Xanthobacter flavus* DT8. Environ. Sci..

[41] Nam J.-H., Ventura J.-R.S., Yeom I.T., Lee Y., Jahng D. (2016). Structural and kinetic characteristics of 1,4-dioxane-degrading bacterial consortia containing the phylum TM7. J. Microbiol. Biotechnol..

[42] Guan X., Liu F., Wang J., Li C., Zheng X. (2018). Mechanism of 1,4-dioxane microbial degradation revealed by 16S rRNA and metatranscriptomic analyses. Water Sci. Technol..

[43] Inoue D., Tsunoda T., Sawada K., Yamamoto N., Saito Y., Sei K., Ike M. (2016). 1,4-dioxane degradation potential of members of the genera *Pseudonocardia* and *Rhodococcus*. Biodegradation.

[44] Masuda H., McClay K., Steffan R.J., Zylstra G.J. (2012). Biodegradation of tetrahydrofuran and 1,4-dioxane by soluble diiron monooxygenase in *Pseudonocardia* sp. strain ENV478. J. Mol. Microbiol. Biotechnol..

[45] Sales C.M., Grostern A., Parales J.V., Parales R.E., Alvarez-Cohen L. (2013). Oxidation of the cyclic ethers 1,4-dioxane and tetrahydrofuran by a monooxygenase in two *Pseudonocardia* species. Appl. Environ. Microbiol..

[46] Li M., Mathieu J., Liu Y., Van Orden E.T., Yang Y., Fiorenza S., Alvarez P.J.J. (2014). The abundance of tetrahydrofuran/dioxane monooxygenase genes (*thmA/dxmA*) and 1,4-dioxane degradation activity are significantly correlated at various impacted aquifers. Environ. Sci. Technol. Lett..

[47] He Y., Mathieu J., Yang Y., Yu P., Silva M.L.B.D., Alvarez P.J.J. (2017). 1,4-dioxane biodegradation by *Mycobacterium dioxanotrophicus* PH-06 is associated with a group-6 soluble di-iron monooxygenase. Environ. Sci. Technol. Lett..

[48] Sales C.M., Mahendra S., Grostern A., Parales R.E., Goodwin L.A., Woyke T., Nolan M., Lapidus A., Chertkov O., Ovchinnikova G. (2011). Genome sequence of the 1,4-dioxane-degrading *Pseudonocardia dioxanivorans* strain CB1190. J. Bacteriol..

[49] Basak B., Chakraborty S., Bhunia B., Dey A., Pramanik K., Patra J.K. (2014). Microbial remediation of recalcitrant aromatic compounds. Industrial & Environmental Biotechnology.

[50] Li M., Liu Y., He Y., Mathieu J., Hatton J., DiGuiseppi W., Alvarez P.J.J. (2017). Hindrance of 1,4-dioxane biodegradation in microcosms biostimulated with inducing or non-inducing auxiliary substrates. Water Res..

[51] Nahar N., Alauddin M., Quilty B. (2000). Toxic effects of toluene on the growth of activated sludge bacteria. World J. Microbiol. Biotechnol..

