# Supplementary material for: Isolation and Characterization of Novel Bacteria Capable of Degrading 1,4-Dioxane in the Presence of Diverse Co-Occurring Compounds
Source: Microorganisms. 2021 Apr 21;9(5):887. doi: 10.3390/microorganisms9050887 (PMC8143092; doi:10.3390/microorganisms9050887)
Supplement: Supplementary file 1 [file microorganisms-09-00887-s001.zip › microorganisms-1192970-supplementary.pdf]

Supplementary Materials

Isolation and Characterization of Novel Bacteria Capable of Degrading 1,4-Dioxane in the Presence of Diverse Co-occurring Compounds

Tanmoy Roy Tusher [1,2], Takuya Shimizu [1], Chihiro Inoue [1] and Mei-Fang Chien [1,*]

[1] Graduate School of Environmental Studies, Tohoku University, Sendai 980-8579, Japan; trtusher.esrm@gmail.com (T.R.T.); takuya.shimizu.p3@dc.tohoku.ac.jp (T.S.); chihiro.inoue.b1@tohoku.ac.jp (C.I.)

[2] Department of Environmental Science and Resource Management, Mawlana Bhashani Science and Technology University, Santosh, Tangail-1902, Bangladesh

* Correspondence: meifangchien@tohoku.ac.jp; Tel.: +81-022-795-7404

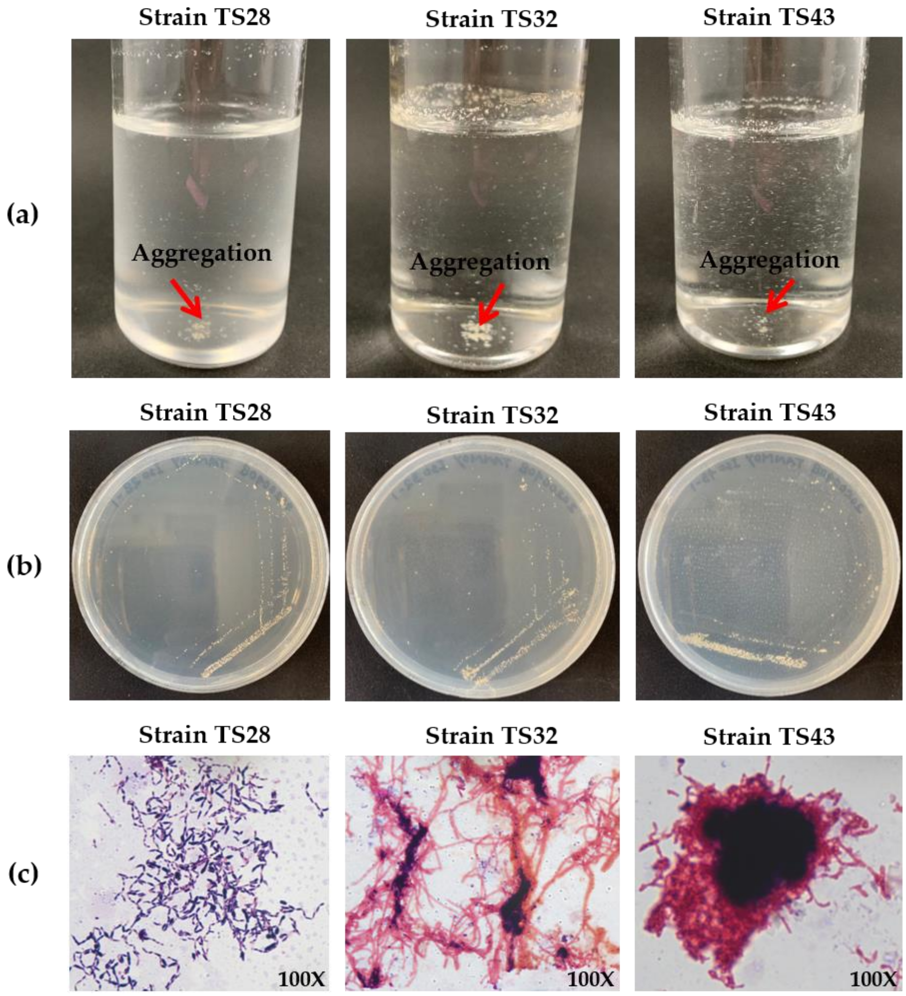

Figure S1. (**a**) Photographs of the isolated 1,4-D-degrading strains forming aggregation in the liquid medium (MSM) supplemented with 1,4-D, (**b**) Colonies of isolated strains on MSM agar plate supplemented with 1,4-D, and (**c**) Gram staining of the isolated strains.

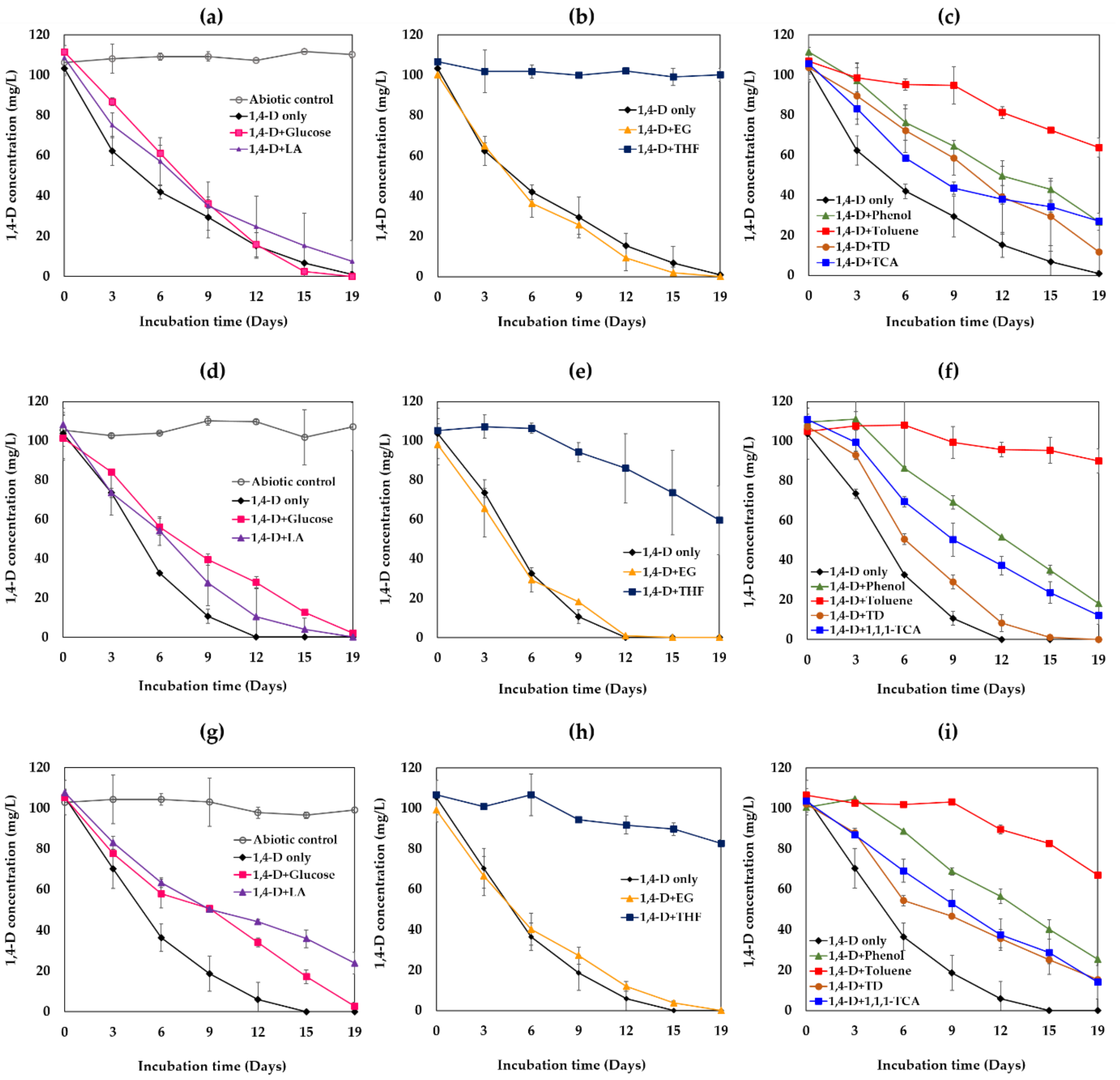

Figure S2. Degradation of 1,4-D (100 mg/L) in the presence of additional carbon sources (100 mg-substrate/L) by isolated strains TS28 (**a**, **b**, **c**), TS32 (**d**, **e**, **f**), and TS43 (**g**, **h**, **i**) under aerobic conditions at 30 °C and 170 rpm. Error bars represent the absolute mean deviations (MD) from the duplicate experiments and may be smaller than the markers. In legends, LA means lactic acid, EG means ethylene glycol, THF means tetrahydrofuran, TD means tetradecane, and 1,1,1-TCA means 1,1,1-trichloroethane.
